# Gutta Percha Bases for Artificial Teeth

**Published:** 1855-04

**Authors:** 


### GUTTA PERCHA BASES FOR ARTIFICIAL TEETH.

The following from the Dental Recorder, gives the only additional information in relation to the use of gutta percha as bases for artificial teeth. We hope some permanent method of coloring will be found out, so that at least beautiful and useful temporary sets may be thus constructed, its adaptation to tender gums would certainly be much more pleasant and easy than any metalic substance.

In a communication received from Dr. D. C. Estes, of Albany, relative to gutta percha bases for artificial teeth, he says, "It is now nearly eight months since I commencedmy experiments with this article, and I have proceeded so far as to insert about one dozen plates, including three entire sets. I first take the raw material, uncompounded with any of the articles usually mixed with it when about to be employed for Dental purposes, and previous to its purification by steam or otherwise. This I purify by treating in hot water and working out the foreign particles. When thus prepared, it is much superior to that which has gone through the melting process for purification, because it requires a much greater heat to affect it.

The first cases put up with this material had the teeth fastened in with the same substance, the base being strengthened by the iron wire as described by Dr. Hill. Although these cases have given satisfaction, still they are liable to objection, as the wire will not always give the desired stability. This objection I have lately overcome in the following manner:—After taking the impression and making the metallic dies in the usual manner, I take tinned sheet iron (such as is used for making tinned ware) and strike up a plate, making it a little smaller every way than it is required to be when completed; then select and arrange the teeth on the plate, with wax, try them in the mouth, and then put the whole in plaster and sand as though for a gold case; remove the wax, line the teeth with the same material as the plate, rivet them firmly, file off and shape the linings with a *coarse* file, so as to leave them as rough as possible, then return the teeth to their bed in the plaster, wash both the linings and the plate under them with muriate of zinc, and proceed to solder by placing small bits of block tin in the crevices and upon the rivets and melting them with the blow pipe. The plate and linings being already tinned over, the solder will flow beautifully and will hold with greater strength than it otherwise would. After this is done, the plate and linings must be made as rough as a burr or coarse file will leave them, and the case then boiled in saleratus water in order to remove the muriate of zinc. Then, with the ordinary puncturing forceps, punch as many holes through the plate as can be made without danger of springing it. This done, take the male die if it is perfect, if not procure one that is, and after thoroughly oiling the surface, take the gutta percha, purified as above and rolled into sheets about the thickness of gold plate, soften it by heat and apply it to the die, pressing it with the fingers wet in warm water to every part intended to be covered by the plate. When this is done, the gutta percha surface must be again softened by a dry heat, as must also the plate of the teeth, the latter must then be placed upon the die covered with the base and pressed quickly and firmly upon it before either the base or plate have time to cool; sometimes it is necessary to repeat the heatings in order to insure success. The plate must be held in position until the base is properly hardened. Then, with a knife blade (which must be heated in the blaze of the lamp from time to time) trim off the superfluous gutta percha, and pack it in between the teeth, filling up all crevices, and in front imitating the form of the gum or alveolus as much as is required to restore the contour of the face; this last can be more perfectly accomplished in this form of work than with any other with which I am acquainted. After this is finished, the plate must be again warmed, another sheet of gutta percha, also warm, pressed firmly upon its lingual surface so as to touch and unite with the base at the opposite surface of the plate, the points of union being the numerous spots of gutta percha protruding through the holes previously punched through the plate. The last layer of gutta percha should be sufficiently broad to allow of its being carried over the linings of the teeth, so as to protect them and the solder from the action of the fluids of the mouth. At this stage of the work a small strip of the gum color should be heated and applied to the front part of the base, so as to imitate the gum as closely as possible. After this, the case should be allowed to cool, and then removed from the die and trimmed with the heated knife blade, care being taken to have every portion of the plate protected by the gutta percha. This completes the work, and if due care has been taken the result will be a beautiful set of teeth, with an accuracy of fit that cannot be possibly attained in any other way, the suction often being so strong as to cause inconvenience in removal from the mouth."

Dr. E. states that he has lately combined other materials with gutta percha, viz:—"equal parts of very fine gypsum, quick lime, and whiting," using as much of the ingredients as can be mixed in without causing the gutta percha to crumble. He further says that Hill's Stopping is the best material known for this style of work, but the expense is such as to preclude all possibility of its being employed in making cheap sets of teeth. He offers to give up experimenting in this direction, provided Dr. H. will reduce the price of his preparation.

Dr. E. goes on to state that the gum color he uses will not stand, and hopes some one possessing greater facilities will give attention to this point, and make known the result.

---

### WAX FOR IMPRESSIONS.

To toughen wax for impressions, melt with one-sixteenth to one eighth part raw white turpentine, according to the temperature of the weather.—*Forcep.*

